# Lessons from history: morbidity of cold injury in the Royal Marines during the Falklands Conflict of 1982

**DOI:** 10.1186/2046-7648-2-24

**Published:** 2013-08-08

**Authors:** Mike Tipton

**Affiliations:** 1 Applied Physiology, Extreme Environments Laboratory, Department of Sport & Exercise Science, University of Portsmouth, Portsmouth, PO1 2ER, UK

## Abstract

Some themes echo through history, the protection provided to our troops is again topical; in 1982 it almost determined the outcome of the Falkland’s campaign. Thirty years on, Frank Golden (who I first met in April of 1982 just after watching the Falkland’s Task Force sail out of Portsmouth) can now tell the story of cold injury in the Royal Marines during the Falklands Conflict (Extreme Physiol Med 2: 23, 2013). It is a fascinating account of hardship, physical endurance, bravery, physiology, pathophysiology, first class scientific operational support and history.

## 

Golden et al. [1] remind us that cold injury is a recurrent theme, playing its part in determining the outcome of conflicts throughout recorded history (see [1] Table one). The difficulty of protecting, particularly the feet, of men operating in the marshy bogs, rocks and barren mountain slopes of the Falklands in the wet-cold of a southern hemisphere winter becomes apparent; with none of the 46 different types of boot worn offering superior protection. It is unsurprising that the combination of hunger, fatigue and cold wet conditions underfoot resulted in cold injures. That 64% suffered cold injuries is surprising, and helps explain why cold injury influenced operational decisions [2].

Even asymptomatic Falkland’s veterans, when tested by Dr Golden soon after the conflict, often showed physiological evidence of mild cold injury (e.g. early and prolonged cold-induced vasoconstriction - “cold hypersensitivity”). Under normal circumstances, this level of injury could be acquired in training or through participation in outdoor pursuits and, although not diagnosed at the time, increase the subsequent risk of cold injury in cold environments. This aspect remains an issue in the UK Armed Forces to this day. Having noted the potential importance of past history of cold injury for subsequent cold injury, Dr Golden’s paper also highlights the fact that in the field, during a conflict in severe conditions, some of the subtle relationships that we manage to finesse in the laboratory and in less severe environments are overwhelmed and become irrelevant.

Those of us who have undertaken field work, or conducted questionnaire surveys cannot fail to be impressed by the amount of data and response rate (78%) Dr Golden and his co-authors managed to acquire from those returning from war; this is a testament to the research team and the armed forces personnel involved. At the time of the conflict Dr Golden was a Surgeon Commander and head of Survival and Thermal Medicine at the Institute of Naval Medicine with, among other things, the responsibility of providing medical support for conditions such as Non-freezing cold injury (NFCI). Time, the number of casualties and the growing operational significance of cold injuries, necessitated a fast response, and having collected the data reported in his paper, Dr Golden set up the UKs first cold injuries clinic, including the tests described in his paper; based on the examination of the neurovascular responses of the injured. Surgeon Commander Golden went on to become Surgeon Rear Admiral Golden OBE, PhD and despite his pioneering work in this and other areas, NFCI has remained a recurrent problem for the military and many other occupational and leisure groups. Whilst freezing cold injury (“frostbite”) is comparatively well understood, NFCI, despite its prevalence, operational significance and potential for resulting in lifelong problems of cold sensitivity, pain and hyperhidrosis, remains poorly understood; its cause, pathophysiology and a definitive diagnostic test for the condition all remain elusive [3].

Finally, a general comment about publication, despite its interest and importance, many journals would not have published Dr Golden’s paper, wanting more than the 30 year-old data could provide, or finding it difficult to categorise. In my view, it is a credit to EPM that it will consider, meticulously review, assist and publish a paper that has undoubted value and that recognises its limitations. Having said this, the editorial team at EPM did debate whether Dr Golden’s paper was a scientific study, an historical account, a case study, a review or a EPM “Career Perspective”. The truth is: it is all of these.

## References

[B1] Golden FSC Francis TJR Gallimore D Pethybridge RJ Lessons from history: morbidity of cold injury in the royal marines during the Falklands conflict of 1982 Extreme Physiol Med 2013 2 23 1 August 2013 10.1186/2046-7648-2-23 PMC3750324 24070118

[B2] Thompson J 3 Commando Brigade in the Falklands: No Picnic 1985 Yorkshire, UK: Pen & Sword Books

[B3] Eglin CM Golden FSC Tipton MJ Cold sensitivity test for individuals with non-freezing cold injury: the effect of prior exercise Extreme Physiol Med 2013 2 16 1 May 2013 10.1186/2046-7648-2-16 PMC3710088 23849038

